# Strain sonoelastography in asymptomatic individuals and individuals with knee osteoarthritis: an evaluation of quadriceps and patellar tendon

**DOI:** 10.1007/s00296-022-05184-3

**Published:** 2022-08-17

**Authors:** Diane M. Dickson, Stephanie L. Smith, Gordon J. Hendry

**Affiliations:** 1 Research Centre for Health, Department of Podiatry and Radiography, School of Health and Life Sciences, Glasgow Caledonian University, 70 Cowcaddens Road, Glasgow, G4 0BA UK; 2 Pain Centre Versus Arthritis, Academic Rheumatology, Injury Recover and Inflammation Sciences, School of Medicine, University of Nottingham, Clinical Sciences Building, Nottingham, NG5 1PB UK

**Keywords:** Elastography, Sonoelastography, Tendon, Knee, Osteoarthritis

## Abstract

An advanced ultrasound imaging technique, sonoelastography (SE) is used to evaluate tissue elasticity. To determine SE potential to detect pathological-related changes, and characteristics related to tendon pathology we aimed to (1) compare quadriceps and patellar tendon findings in individuals with knee osteoarthritis (KOA) and asymptomatic older adults (AC), and (2) explore associations between SE, participant characteristics (age, BMI, and leg circumference) and KOA status. 84 participants (47; KOA and 37; asymptomatic older adults) underwent SE examination of quadriceps (distal) and patellar (distal, proximal) tendon in a supine position with the knee bent at 30°. Colour score (CS) and Elasticity Ratio (ER) analysis were performed by a blinded experienced operator using Esaote Mylab 70 XVG Ultrasound equipment. Significantly reduced elasticity in the distal quadriceps (median (IQR) 2(2), 3(1), *p* = 0.033 for KOA and AC, respectively) and proximal patellar (3(1), 3(0), *p* = 0.001) tendons and more elastic distal patellar (1.50 (0.55), 1.87 (0.72), *p* = 0.034) tendons were observed in the KOA group. Significant associations) were identified between SE and participant BMI (Rs = − 0.249–0.750, *p* < 0.05) and leg circumference (Rs = − 0.260–0.903, *p* < 0.05). Age, BMI and KOA status, were independent explanatory variables of SE CS findings at the distal quadriceps tendon patellar tendon, proximal patellar tendon and distal patellar tendon, explaining 66%, 81% and 64% of variance, respectively. Age, BMI and KOA status were independent explanatory variables of SE ER findings at the distal patellar tendon explaining 19% of variance. Potentially clinically relevant altered tendon stiffness were observed between individuals with KOA and asymptomatic controls. Key KOA risk factors and participant characteristics explained variance in tendon stiffness. Findings provide context for future studies to investigate the potential for targeted SE detected early clinical management based on associated participant characteristics.

**Supplementary Information:**

The online version contains supplementary material available at 10.1007/s00296-022-05184-3.

## Introduction

Knee osteoarthritis (KOA) is a highly prevalent global health burden, and preventative strategies are a key health priority [1, 2]. The aetiology of KOA remains under investigation, however, multiple risk factors are known to contribute to disease onset and progression. These include age, female sex, genetics, previous injury, biomechanical factors and obesity [1, 3, 4]. Quadriceps muscle weakness has also been associated with radiographic KOA [5, 6] and pain [7, 8], negatively impacting on physical function and the ability to participate in daily activities [9]. Furthermore, knee extensor weakness has been shown to increase the risk of worsening symptoms and functional deterioration [10]. Knee extensor weakness is considered a risk factor for KOA [4]. A recent systematic review found increased odds (30%) of symptomatic and radiographic KOA in individuals with knee extensor muscle weakness, however, it was acknowledged that quality of evidence was low [11]. However, targeting modifiable risk factors such as maintaining appropriate quadriceps muscle strength and improving lifestyle factors such as exercise and weight loss can help reduce the risk of KOA [12, 13]. Knee extensor tendon degeneration has been found to predate the onset of KOA by 2 years [14] which may be an important subclinical opportunity for intervention. Pathological changes in knee joint tendons have been demonstrated in individuals with KOA, with tendon changes associated with previous knee joint injury, including anterior cruciate ligament, predicting knee osteoarthritis [15].

Knee joint function requires the maintenance of extensor apparatus, including important peri-articular knee tendon structural integrity. Tendon elasticity is a mechanical property representing the ratio of force applied to a tendon and its elongation in response to force. Strain sonoelastography (SE) is a technique widely used in breast, thyroid, liver and prostate imaging, often used to characterise lesions and assess tissue stiffness [16, 17]. Within musculoskeletal imaging, tendon elasticity can be evaluated by SE through deformation of the tendon under an applied external stress, where small strain represents a stiffer tendon, and large strain corresponds to more elastic tissue. Common SE measurement methods include colour scoring (CS), where a colour-coded elastogram represents different magnitudes of relative tissue strain. Elasticity ratio (ER) can also be employed to demonstrate elastic contrast through semi-quantitative assessment of two defined regions of interest (ROI) [18].

KOA is characterised as a whole joint disease, which manifests as structural changes of varying degrees throughout articular cartilage, subchondral bone, capsule, synovial membrane, ligaments and peri-articular muscles [4]. SE has potential for the detection and monitoring of tendon alterations [19], therefore, an understanding of knee tendon elasticity changes in KOA may be of benefit to inform early intervention such as targeted rehabilitation. A recent systematic review reports high variability in the quality of many existing knee tendon SE studies [20]. Our previous work provides evidence of reliability of a standardised SE technique when performed and interpreted by an experienced operator. With further standardisation of SE methods and a future research focus on predictive validity and diagnostic accuracy, this method may provide a quick and reliable method of detecting any early tendon changes associated with KOA and/or other tendon pathologies.

Tendon elasticity can influence muscle power and transmit forces, therefore, tendon health is vital for effective muscle to tendon interfaces [21]. Knee tendon are subject to large tensile forces and sites of stress where the tendon attaches to bone [22]. There is potential for cumulative micro-trauma from injury and disease processes; leading to tendon pathology, with the potential to impair force transmission and muscle and knee function [23]. Understanding tendon elasticity and pathological changes related to KOA, may not only be important for early identification of KOA, and targeted interventions, it may identify potential barriers to rehabilitation. Combined with determining associations with participant characteristics may help identify potential risk factors, and characteristics which may explain variance in ultrasound SE.

This cross-sectional comparative exploratory design study seeks to investigate quadriceps and patellar tendon elasticity in KOA through (1) evaluation of differences between SE measures of individuals with KOA and an asymptomatic older adult control population, (2) quantification of quadriceps and patellar tendon elasticity using SE measures in individuals with KOA, (3) association of quadriceps and patellar tendon SE and participant characteristics (age, sex, BMI and leg circumference) and (4) association of quadriceps and patellar tendon SE, with participant characteristics and KOA status.

## Materials and methods 

### Sample population

Individuals aged 40 years or over with uni-/bi-lateral doctor diagnosed KOA were recruited from rheumatology clinics; general practitioner (GP) practices, an active ageing database of older adults who had volunteered to be contacted for research, and the general adult population using a local newspaper advert. KOA diagnosis was determined by a combination of knee radiograph report (rheumatology clinic recruits), KOA International Classification of Diseases (IDC-10) codes (GP recruits), and participant expressed GP consultation confirmation of KOA (active ageing database and newspaper advert recruits). All participants with diagnosed KOA underwent email/telephone screening to ensure they had knee pain most days in the past month and stiffness in the morning lasting less than 30 minutes. An asymptomatic healthy control population was recruited from friends, family, active ageing database of older adults and University staff, and were contacted via telephone/email to ensure they were free from KOA symptoms prior to enrolment.

Participants were excluded if they had any known neuromuscular skeletal injury or disease; knee surgery in the past year; knee replacement in the test knee; steroid injections in the past 3 months or severe co-morbidity. Asymptomatic individuals were excluded if they had any history of KOA or chronic or stable knee pain in the past 3 months; previous knee surgery, had a history of autoimmune or connective tissue disorder, or if they were in receipt of oestrogen or steroid medication due to previous association with tendon abnormalities [24]. Due to participant comorbidities and reflective of population groups, the KOA group were not excluded on the basis of oral hormone or steroid use (*n* = 2). Written informed consent was obtained from all participants included in the study. The study was approved by the West of Scotland research ethics committee (13/WS/0146) and Glasgow Caledonian University (HLS12/86, HLS/PSWAHP/16/203) and was carried out within the imaging suite at Glasgow Caledonian University.

### Equipment

Participants were examined using ultrasound (US) equipment, Esaote Mylab 70 XVG, version EVO 13.60 M with multi frequency linear array transducer (LA523, L4-13 MHz). A standard measurement tape was used to measure leg circumference in centimetre (cm), at the level of mid-pole patellar. Height was measured using a stadiometer (cm) and body mass was measured using standard mechanical scales (kg).

### Ultrasound protocol

**Fig. 1:**
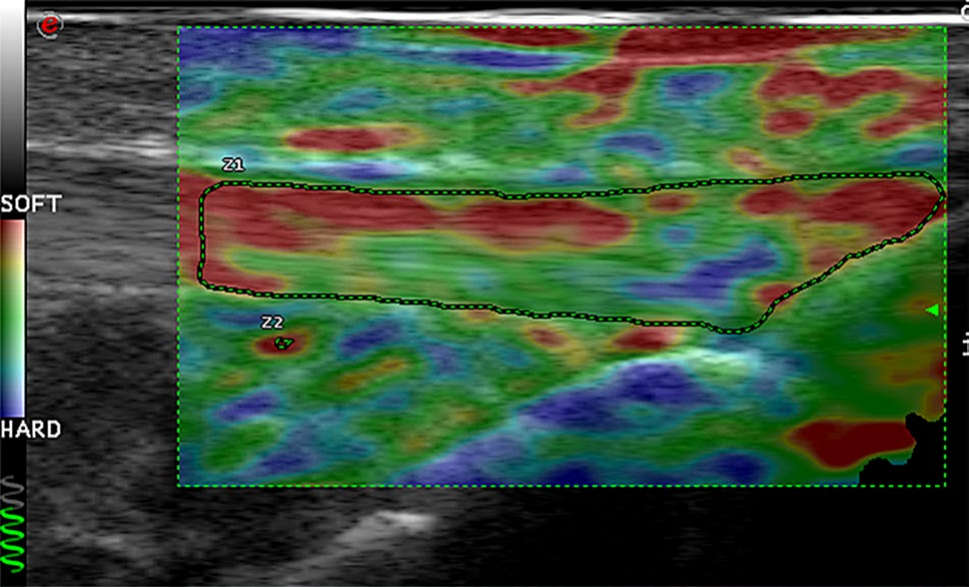
Standardised elastogram landmark and elasticity ratio measurement method for DPT [32]

All scans and image analysis were performed by a blinded experienced operator [DMD] with 12 years of US experience and SE experience of > 50 participant examinations. Participants self-reported principally symptomatic (knee osteoarthritis group [KOA]), and dominant (asymptomatic control [AC] groups’) lower limb were scanned in a lying position with the knee supported in 30° of flexion, in line with current imaging guidance [25] following 30 min of seated rest. Using B-mode US, the distal quadriceps tendon (DQT) was located in longitudinal orientation using the base of patella as the distal landmark, and an elastogram performed for a minimum 5 s using previously published optimal settings [26]. The proximal patellar tendon (PPT) was identified using the patella as a superior landmark border and the distal patellar tendon (DPT) region by the tibial tuberosity, where elastograms were applied. A representative static image which demonstrated sufficient stress as demonstrated by the equipment quality indicator was selected to perform image analysis. CS was visually graded using a similar three-point scale employed in previous studies [27–29], stiff = 1, intermediate = 2, and soft = 3 depicted by SE colour map. Elasticity ratio (ER) measures were performed using corresponding reference sites previously employed in published studies (quadriceps tendon = pre-femoral fat pad and patellar tendon = Hoffa’s fat pad) [30, 31], a small fixed size reference ROI of 1 mm was positioned within homogenous fat pad tissue. An anatomical site ROI (DQT, PPT and DPT) was freehand traced with US machine tracker-ball, and an ER value was automatically calculated within the ElaXto equipment package, and recorded (see Fig. 1). A fuller description of the SE protocol and evaluation is reported elsewhere [32].

### Statistical analysis

Normality was assessed using skewness and kurtosis. Descriptive statistics were used to present participant characteristics, expressed as means (standard deviations) or absolute (number, *n*) and relative (percentages, %) frequencies, with elasticity values expressed by median and interquartile ranges. Knee (OA) and asymptomatic control group differences in participant characteristics (age, sex, BMI, leg circumference) and elasticity values were evaluated using Mann–Whitney *U* tests. Additionally, sex-stratified KOA group differences were evaluated. Spearman’s correlations were performed to assess associations between elasticity values and participant characteristics. The correlation statistical threshold levels for interpretation were defined as ≤ 0.29, small; 0.30–0.49, medium and ≥ 0.50 large [33]. Logistic regression (with odds ratios, [OR]) was used to evaluate the relationship between CS (dependent variable) and participant characteristics (age, sex, BMI, leg circumference and KOA status). CS values stiff and intermediate were combined and recoded (1) to represent stiff tendon, CS value soft, was reassigned 0 to enable regression analysis and to identify the reduced elasticity tendon categories as the research focus. A three-step model was performed through a bivariate step followed by backwards, stepwise group multivariate logistic regression. Bivariate associations were examined between covariate and the outcome (CS), potential covariates *p* > 0.20 were rejected, and *p* < 0.20 taken forward into backward stepwise group modelling. Multiple linear regression was performed to evaluate the relationship (*β*; standardised coefficient Beta) between ER values and participant characteristics age, sex, BMI, leg circumference and KOA status. *T* tests assessed differences between missing and complete data. Two-tailed statistical significance was defined as *p* ≤ 0.05. Statistical analysis were performed using SPSS, version 24 [34].

## Results

A total of 84 individuals were included in this study (Table 1), 47 (56%) with previously diagnosed KOA (KOA, 32 females, 15 males); most symptomatic knee, left *n* = 22, right *n* = 25 and 37 (44%) asymptomatic controls (AC, 26 females, 11 males); self-reported dominant lower limb, left *n* = 12, right *n* = 25. The KOA population were older (*p* = 0.029) with higher BMI (*p* = 0.001), and greater leg circumference (*p* = 0.001), compared to the AC group (Table 1). There were no differences in test leg distribution between groups (*p* = 0.185).

**Table 1:** Participant characteristics

Characteristic	KOA group *n* = 47	AC group *n* = 37	*p*
Female/male n(%)	32 (68)/15 (32)	26 (70)/11 (30)	0.831
Age years	61.8 (8)	58.3 (7.5)	**0.029**
BMI kg/m^2^	29.8 (5.8)	25.7 (3.1)	**0.001**
Leg circumference cm^a^	41.6 (4.1)	38.3 (2.6)	**0.001**
Test leg left/right n (%)	22 (47)/25 (53)	12 (32)/25 (68)	0.185

Data presented as mean (standard deviation) or *n* (%)

*BMI* Body Mass Index, *KOA* knee osteoarthritis group, *AC* asymptomatic control group

^a^Leg circumference data based on *n* = 36 for KOA group and AC group *n* = 29. Bold text indicates *p* < 0.05 for Mann–Whitney U test statistic

### Colour score

The distal quadriceps tendon (DQT, *p* = 0.033) and proximal patellar tendon (PPT, *p* = 0.001) were significantly less elastic in KOA group participants, compared to the AC group (Tables 2, 3). No significant difference was observed between KOA and AC groups at the distal patellar tendon (DPT; *p* = 0.646, Table 3). A significant sex difference was observed within the KOA group for CS measures of the DQT (*p* = 0.024) and DPT (*p* = 0.022) with females demonstrating less elastic tendon compared to males (Table 4).

**Table 2:** Description of tendon site elasticity across all groups

Site	Colour score			Elasticity ratio		
	Total population	KOA group	AC group	Total population	KOA group	AC group
	Median (IQR)	Median (IQR)	Median (IQR)	Median (IQR)	Median (IQR)	Median (IQR)
DQT	2 (2)	2 (2)	3 (1)	2.23 (1.16)	2.44 (1.40)	2.03 (0.73)
PPT	3 (0)	3 (1)	3 (0)	1.43 (0.61)	1.53 (0.84)	1.37 (0.22)
DPT	3 (1)	3 (1)	3 (1)	1.59 (0.55)	1.50 (0.43)	1.87 (0.72)

*KOA* knee osteoarthritis group,* AC* asymptomatic control group,* DQT* distal quadriceps tendon,* PPT* proximal patellar tendon,* DPT* distal patellar tendon,* CS* colour score,* ER* elasticity ratio,* IQR* interquartile range

*Total population CS; n* = *84, ER DQT; n* = *77, ER PPT; n* = *57, ER DPT; n* = *59, KOA CS; n* = *47, ER DQT; n* = *40; ER PPT; n* = *39, ER DQT; n* = *41, OC CS; n* = *37, ER DQT; n* = *17, ER PPT; n* = *18, ER DPT; n* = *18*

**Table 3:** Difference in elasticity between knee osteoarthritis group and older control group

Tendon site	Colour score						Elasticity ratio					
	KOA group		AC group		Mann–Whitney* U*	*p*	KOA group		AC group		Mann–Whitney* U*	*p*
	*n*	Mean rank	*n*	Mean rank			*n*	Mean rank	*n*	Mean rank		
DQT	47	37.78	37	48.50	647.5	**0.033**	40	31.25	17	23.71	250	0.166
PPT	47	36.74	37	49.81	599.0	**0.001**	39	31.33	18	23.94	260	0.118
DPT	46	41.10	37	43.12	809.5	0.646	41	26.85	18	37.17	240	**0.034**

*KOA* knee osteoarthritis group,* AC* asymptomatic control group,* n* number,* DQT* distal quadriceps tendon, *PPT* proximal patellar tendon,* DPT* distal patellar tendon, bold* text indicates Mann Whitney U test statistic p* < *0.05*

**Table 4:** Difference in elasticity between sex for the knee osteoarthritis group

Tendon site	Colour score						Elasticity ratio					
	KOA group female		KOA group male		Mann–Whitney* U*	*p*	KOA group female		KOA group male		Mann–Whitney* U*	*p*
	*n*	Mean rank	*n*	Mean rank			*n*	Mean rank	*n*	Mean rank		
DQT	32	21.14	15	30.10	148.5	**0.025**	28	22.39	12	16.08	115.0	0.118
PPT	32	23.03	15	26.07	209.0	0.402	27	22.24	12	14.96	101.5	0.066
DPT	31	20.84	15	29.00	150.0	**0.022**	28	22.13	13	18.58	150.5	0.382

*KOA* knee osteoarthritis group,* n* number,* DQT* distal quadriceps tendon,* PPT* proximal patellar tendon, *DPT* distal patellar tendon,* p* values are based, bold text indicates Mann Whitney U test statistic *p* < 0.05

### Elasticity ratio

There was no statistically significant difference in DQT and PPT elasticity (*p* = 0.166, *p* = 0.118, Table 3). The KOA group DPT was significantly more elastic than the AC group (*p* = 0.034, Table 3). ER did not differ between sexes within the KOA group (Table 4).

### Association of elasticity values with participant characteristics

#### Age

There was no association (*Rs*; − 0.322–0.271, *p* > 0.05) between age and elasticity measures (Table 5). There remained no association when stratified by population group. Within the KOA group, both sexes demonstrated a similar direction of increased tendon elasticity with age at the level of the DQT (Supplementary Figure S1).

**Table 5:** Association between elasticity values and participant characteristics

Characteristic	Population	*n*	Colour score			Elasticity ratio		
			CS DQT *R*_*s*_ sig	CS PPT *R*_*s*_ sig	CS DPT *R*_*s*_ sig	ER DQT *R*_*s*_ sig	ER PPT *R*_*s*_ sig	ER DPT *R*_*s*_ sig
Age	KOA AC All	CS = 47 ER = 39–41 CS = 37 ER = 18 CS = 84 ER = 57–59	0.089 *p* = 0.552 0.019 *p* = 0.909 0.002 *p* = 0.989	− 0.083 *p* = 0.580 − 0.011 *p* = 0.948 − 0.117 *p* = 0.290	− 0.062 *p* = 0.680 − 0.094 *p* = 0.579 − 0.078 *p* = 0.486	− 0.189 *p* = 0.244 0.271 *p* = 0.293 0.012 *p* = 0.927	0.086 *p* = 0.603 0.130 *p* = 0.608 0.159 *p* = 0.237	0.075 *p* = 0.642 − 0.322 *p* = 0.192 − 0.130 *p* = 0.328
BMI	KOA AC All	CS = 47 ER = 39–41 CS = 37 ER = 18 CS = 84 ER = 57–59	− 0.030 *p* = 0.843 − 0.288 *p* = 0.084 − 0.190 *p* = 0.083	− 0.281 *p* = 0.055 − 0.017 *p* = 0.921 **− 0.322 *p* = 0.003**	− 0.223 *p* = 0.136 **− 0.348 *p* = 0.035** **− 0.249 *p* = 0.023**	0.194 *p* = 0.230 0.115 *p* = 0.659 0.219 *p* = 0.102	0.234 *p* = 0.151 0.118 *p* = 0.642 **0.277 *p* = 0.037**	0.258 *p* = 0.103 **0.750 *p* < 0.001** 0.189 *p* = 0.151
Leg circumference	KOA AC All	CS = 36 ER = 31–32 CS = 29 ER = 11 CS = 65 ER = 42–43	− 0.104 *p* = 0.547 − 0.293 *p* = 0.123 **− 0.260 *p* = 0.037**	**− 0.457 *p* = 0.005** c **− 0.477 *p* < 0.001**	− 0.264 *p* = 0.125 − 0.189 *p* = 0.327 − 0.220 *p* = 0.080	0.153 *p* = 0.403 0.095 *p* = 0.782 0.228 *p* = 0.142	0.242 *p* = 0.189 0.134 *p* = 0.695 0.296 *p* = 0.057	0.199 *p* = 0.274 **0.903 *p* < 0.001** 0.109 *p* = 0.486

*KOA* knee osteoarthritis group, *AC* asymptomatic control group, *BMI* Body Mass Index, *CS* colour score, *ER* elasticity ratio, *DQT* distal quadriceps tendon, *PPT* proximal patellar tendon, *DPT* distal patellar tendon, *n* number, *R*_*s*_ Spearman’s correlation statistic, *sig* statistical significance, *c* constant, bold text indicates *p* < 0.05

#### BMI

Within the total population, a higher BMI was associated with less elastic (CS) PPT and DPT (stiff coded 1, intermediate 2 and soft, 3, Table 5) and PPT ER (*Rs* = 0.277, *p* = 0.037, Table 5). Stratified by groups, CS and ER DPT elasticity reduced with increasing BMI (CS; *Rs* = − 0.348, *p* = 0.035, ER; *Rs* = 0.75, *p* < 0.001, Table 5) in the AC group only. In individuals with KOA, there was a trend (non-significant) towards higher BMI with reduced ER tendon elasticity at all regions (Supplementary Figure S2). There were no further associations across groups (*p* > 0.05; Table 5).

#### Leg circumference

Within the total population, greater leg circumference had a weak to moderate association with reduced tendon elasticity (CS) DQT and PPT measures (Table 5). Increased leg circumference was strongly associated with increased ER in the AC group (*Rs* = 0.903, *p* < 0.001, Table 5). The same association was observed for CS PPT in the KOA group (*Rs* = − 0.457, *p* = 0.005, Table 5).

### Association of KOA with participant characteristics and tendon elasticity 

#### Variable selection

Participant characteristics; sex, age, BMI, leg circumference and KOA status were considered for regression analysis, with CS elasticity values assigned as the dependent variable (0 = no and 1 = yes). Collinearity testing determined BMI and leg circumference were strongly positively correlated (*Rs* = 0.823, *p* < 0.001), therefore, leg circumference was excluded as a covariate. Model 1 included BMI and age, model 2; BMI, age and sex, and model 3, BMI, age, sex and KOA status.

#### Logistic regression

Models 2 and 3 were statistically significant at the DQT region, where 63–66% of tendon elasticity variance were explained by participant characteristics. Sex was the greatest contributor at the DQT region, where female sex associated with reduced elastic tendon at an odds ratio (OR) of 3.548–3.621 (*p* ≤ 0.013, Table 6). The same independent variables accounted for 81–82% of PPT and 64–68% of DPT variance. KOA status as an independent variable marginally failed to reach statistical significance within model 3 at the PPT region (OR; 5.157, *p* = 0.053), however, BMI was significantly associated with a reduction in tendon elasticity (OR; 1.151, *p* = 0.019, Table 6). BMI (OR; 1.132, *p* = 0.025) was significantly associated with reduced tendon elasticity at the DPT (Table 6).

**Table 6:** Logical regression analysis of colour score elasticity values and participant characteristics

Model	CS DQT				CS PPT				CS DPT			
	%	EXP (B)	CI	sig	%	EXP (B)	CI	sig	%	EXP (B)	CI	sig
1. BMI	60	1.097	0.997–1.207	0.059	*81	1.213	1.083–1.357	**0.001**	65	1.118	1.015–1.232	**0.024**
Age,		1.007	0.952–1.064	0.814		1.062	0.980–1.151	**0.139**		1.018 1.123	0.957–1.082	0.573
2. BMI	*63	1.105	0.998–1.222	0.055	*82	1.211	1.082–1.355	**0.001**	*68	1.024	1.017–1.240	**0.022**
Age		1.015	0.957–1.075	0.624		1.064	0.982–1.153	**0.618**			0.962–1.090	0.462
Sex,		3.548	1.307–9.638	**0.013**		1.380	0.389–4.896	0.618		3.154	0.992–10.03	0.052
3. BMI	*6	1.084	0.972–1.209	0.145	*81	1.151	1.024–1.293	**0.019**	*64	1.132	1.015–1.262	**0.025**
Age		1.008	0.948–1.070	0.807		1.039	0.958–1.127	0.355		1.027	0.962–1.097	0.420
Sex		3.621	1.320–9.938	**0.012**		1.493	0.411–5.427	0.543		3.137	0.986–9.975	0.053
OA	6	1.625	0.579–4.561	0.356		5.157	0.997–27.22	0.053		0.809	0.266–2.460	0.809

*OA* osteoarthritis status,* BMI* Body Mass Index,* CS* colour score,* DQT* distal quadriceps tendon,* PPT* proximal patellar tendon,* DPT* distal patellar tendon,* n* number,* %* percentage,* EXP (B)* exponential of the coefficients (Odds Ratio),* CI* confidence intervals,* SE* unstandardised standard error,* sig* statistical significance, *Indicates model significance, bold text indicates *p* < 0.05, statistics based on *n* = 84

### Linear regression

Linear regression model 2 was contributed to significantly by BMI (*β*; 0.292, *p* = 0.028) at the DQT, with model 2 explaining 12% of SE variance. However, when KOA was added, BMI was no longer significant (Table 7). BMI (*β*; 0.390, *p* < 0.006) and KOA status were significant independent variables within model 3 at the DPT (0.395, *p* = 0.009), explaining 19% of ER variance (*p* < 0.05, Table 7).

**Table 7:** Linear regression analysis of elasticity ratio values and participant characteristics

Model	ER DQT					ER PPT					ER DPT					
	*R*^2^	*β*	SE	CI	sig	*R*^2^	*β*	SE	CI	sig	*R*^2^	*β*	SE	CI	sig	
1. BMI	0.097	0.303	0.023	0.008–0.099	0.240	0.074	0.192	0.016	− 0.009–0.055	0.149	0.071	0.230	0.011	− 0.002–0.041	0.079	
Age		− 0.710	0.160	− 0.040–0.023	0.590		0.186	0.010	− 0.006–0.03	0.161		− 0.143	0.007	− 0.021–0.006	0.271	
2. BMI	0.123	**0.292**	0.023	0.006–0.097	**0.028**	0.085	0.188	0.016	− 0.009–0.055	0.160	0.077	0.229	0.011	− 0.003–0.041	0.083	
Age		− 0.050	0.016	− 0.037–0.025	0.700		0.199	0.010	− 0.005–0.037	0.139		− 0.013	0.007	− 0.015–0.013	0.924	
Sex		0.161	0.278	− 0.213–0.903	0.220		0.102	0.185	− 0.229–0.514	0.445		0.078	0.124	− 0.174–0.324	0.550	
3. BMI	0.145	0.220	0.025	− 0.012–0.089	0.130	0.095	0.140	0.018	− 0.018–0.053	0.339	*0.189	**0.390**	0.011	0.010–0.055	**0.006**	
Age		− 0.116	0.017	− 0.0480.020	0.414		0.152	0.012	− 0.011–0.035	0.302		− 0.013	0.007	− 0.015–0.013	0.924	
Sex		0.138	0.281	− 0.267–0.859	0.297		0.088	0.188	− 0.254–0.500	0.516		0.115	0.118	− 0.127–0.347	0.357	
OA		− 0.179	0.340	− 1.076–0.288	0.252		− 0.124	0.225	− 0.627–0.275	0.437		**0.395**	0.142	0.103–0.673	**0.009**	

*OA* osteoarthritis status,* BMI* Body Mass Index,* ER* elasticity ratio,* DQT* distal quadriceps tendon,* PPT* proximal patellar tendon,* DPT* distal patellar tendon,* n* number,* %* percentage,* CI* confidence intervals,* R*^*2*^ *R* square, (linear), *β* standardised coefficient Beta*, SE* unstandardised standard error, *sig* statistical significance, *indicates model significance, bold text indicates *p* <* 0.05, statistics based on n* = 57

## Discussion

This study is the first to report the SE characteristics of quadriceps and patellar tendon of individuals with KOA, compared to asymptomatic adult controls. Individuals with KOA had significantly less elastic CS values of the DQT and PPT and more elastic ER measures of the DPT, than asymptomatic older adults. Females with KOA had significantly reduced elasticity (CS values) of the DQT and DPT compared to males with KOA. Additionally, BMI and KOA status were significantly associated at the DPT, explaining 19% of SE variance. These findings provide novel insight into measurable differences between SE quadriceps and patellar tendon elasticity of KOA individuals and asymptomatic individuals, providing context for future investigation.

### KOA comparisons

The ability to detect SE differences of quadriceps and patellar tendon between asymptomatic and KOA individuals may offer earlier opportunity to deliver enhanced rehabilitation strategies. To date, mixed results in relation to superiority of SE techniques exist [35, 36], however, our findings demonstrate potentially clinically important and statistically significant differences between groups in both CS and ER techniques. Nevertheless, recent meta-analyses recognise that SE remains largely unstandardised and further work is required to minimise bias and improve reliability [20].

Reduced DQT elasticity in the KOA population compared to the AC group (*p* = 0.033, Tables 2, 3) may be a particularly valuable finding due to previous association with reduced quadriceps muscle strength and reduced rate of force development in KOA [5, 37], and the functional role of the muscle tendon interface [6]. Recent studies report association of reduced quadriceps tendon elasticity with reduced maximum knee flexion angle [38] and increased quadriceps muscle stiffness in KOA individuals compared to healthy controls [39] which provide further supporting evidence of measurable differences between groups. Furthermore, a reduction in extensor strength in a female population was reported to precede symptomatic knee progression [40]. In the absence of existing literature and lack of understanding of the degenerate extensor mechanism and SE’s role in the detection and monitoring of KOA, the current findings require further longitudinal investigation to determine the clinical significance in the context of early/subclinical disease progression. A recent small-scaled study found no significant change in SE (ER technique) from baseline to 12 weeks following an exercise regime for supraspinatus tendinopathy, despite significantly improved patient reported outcomes [41]. However, over a longer time frame of 40 days, 6 months and 12 months, Achilles tendon (AT) rupture post-operative longitudinal changes were detected by SE and characterised by significantly reduced tendon elasticity than the contralateral untreated AT (ER technique) [42]. It was also noted that the contralateral AT were found to become less elastic over time, likely due to tendon overload during rehabilitation [42]. It is previously reported that adults with KOA are twice as likely to have MRI detected degenerate knee extensor tendons 2 years prior to onset of criteria-based incident KOA disease [14]. This suggests that longitudinal changes over this timeframe may be conceivably detected by SE, however, minimal detectable change in this population warrants investigation.

Female sex and increased BMI are well-documented risk factors in KOA [4, 43]. Correspondingly, we found BMI to be significantly greater in the KOA population (*p* = 0.001). Previous shear wave elastography (using shear wave velocity quantification for tissue elasticity) research has demonstrated that healthy males have significantly more elastic patellar tendons than females, due to higher body mass and male muscle strength [44]. In contrast, another study found no significant differences in patellar tendon elasticity between sex [45]. Our study is the first to identify statistically significant differences in SE quadriceps tendon elasticity between males and females in the KOA population, where females had significantly less elastic CS values of the DQT (*p* = 0.025) and DPT (*p* = 0.022), compared to males (Table 4). Furthermore, female sex was found to be a significant independent variable in logistic regression analysis (OR; 3.548–3.621, *p* ≤ 0.013) for reduced tendon elasticity DQT measures (Table 6). Using widely accessible ultrasound technology [46], future clinical evaluation of SE differences between groups may enable timely and relatively cost effect targeted diagnosis of high-risk populations based on established KOA risk factors to provide improved management of KOA.

Age is a known risk factor in the development of KOA [4]. Despite a statistically significant difference in age groups between the OA and AC group (variance in mean of + 3.5 years for the OA group, *p* = 0.029, Table 1), age was not found to be associated to tendon elasticity within the AC or OA populations (Table 5). Therefore, the observed differences in elasticity values within the OA group may be attributed to other factors, including KOA pathology. When stratified by sex, the OA group tendon elasticity generally reduced with age, except at the DQT, where increased elasticity with age was observed (Supplementary Figure S1). Therefore, although elasticity is comparatively reduced in individuals with OA, an increased elasticity with increasing age at the DQT within the OA group, was apparent (statistically significant in male OA group *r* = − 0.684, *p* = 0.014 only). Tendon properties match the level of muscle performance [47], therefore, reduced quadriceps muscle and joint function observed in KOA may account for this finding. KOA duration and severity was not accounted for within the population, therefore, changing elasticity over disease progression may contribute to this result. Further investigation into tendon elasticity changes over disease course is required to fully understand this finding.

Our study identifies that elasticity values vary across different tendon sites, which may be accounted for by the mechanical role of each specific tendon region. Unlike the DQT and PPT, the DPT was found to be significantly more elastic (ER) in the OA population, compared to asymptomatic older adults (*p* = 0.034, Table 3). Furthermore, ER of the DPT was significantly associated with KOA status (*p* = 0.009, Table 7). Biomechanical and structural joint data were not included in this study and in the absence of existing literature this finding cannot currently help explain the pathophysiological or clinical significance of these findings, which warrant future investigation. A potential theory may be that joint space narrowing, typical of KOA, may contribute a less taut patellar tendon, leading to increased elasticity. Future work is required to explore these findings, however, regardless of cause, there may be an opportunity to detect atypical tendon changes to help manage and improve tendon composition and subsequent joint condition.

The PPT region is most frequently affected by tendinopathy [48], and has a greater role in the transfer of quadriceps muscle strength [49]. Therefore, the PPT may be more vulnerable to pathological alterations, and may contribute to the finding of reduced elasticity in this region within the KOA compared to AC group (Tables 2 and 3). ER of the PPT was not found to be statistically significant within logistic regression modelling but just failed to reach significance within the bivariate model (OR 2.732; 95% CI 0.063–33.546, *p* = 0.06) which may suggest a level of association with KOA classification, although wide confidence intervals as a consequence of small group sample sizes must be acknowledged. Individuals with KOA are at increased risk of injury subsequent to pain, stiffness, imbalance and comorbidities [50–52]. Injury may further exacerbate tendon properties, therefore, the impact of injury and/or KOA pathogenesis may be responsible for significant reduction in quadriceps and patellar tendon elasticity observed at the PPT and DQT. Further investigation is required to determine the significance of these findings.

Previous studies have demonstrated change in tendon size and mechanical properties following resistance training [48, 53]. Recently, a shear wave elastography study reported significant difference between controls and participants with gout [54], further strengthening the evidence of capability of elastography US techniques to determine between control and pathological groups. Yet, a systematic review of 11 elastography studies determined no significant difference between rotator cuff tendons with and without tendinopathy but was able to demonstrate significant difference in cases of adhesive capsulitis [55]. It remains uncertain where SE fits in the clinical setting, however, detection of modifiable tendon properties in KOA and subclinical KOA warrants investigation in future SE studies. It remains challenging to interpret results of studies concerning tendon elasticity alterations due to heterogeneous methods. Further research is required to help develop a greater understanding of the normal and KOA tendon structure and should include both elastography and correlation with clinical symptoms.

### Limitations

To reduce technician variation, all scans were performed by the same operator using the same protocols and equipment, however, there are limitations to this work. An equipment fault led to a reduced number of asymptomatic older adult control ER measurements being included in analysis, a larger number may have provided more statistical power. The exploratory nature of this cross-sectional comparison study provided no requirement for power calculation prior to analysis [56]. However, retrospective sample size calculation using G*power determined that for *p* < 0.05, 80% power and to detect an effect size of at least 0.6 (medium-to-large), 47 participants for a 2 group comparison via Mann–Whitney *U* is required [57]. Therefore, limitations can be considered in comparisons between the KOA (*n* = 47) and AC (*n* = 37) group which were slightly underpowered. Wide odds ratio confidence intervals demonstrated in regression analysis are also considered a limitation that can be attributed to the small sample size. As such, results should be interpreted with caution given the increased risk of type 2 error. Leg circumference measurements were not available for all participants and led to a reduction in *n* for the relative correlation statistics. Statistically significant differences between control and KOA groups were evident, however, closer control group matching may have contributed to more robust comparisons between groups (KOA and AC). Furthermore, AC group participants may have presented with asymptomatic KOA features which could have affected results [58] and the method of diagnosis of KOA was non-standardised across recruitment groups, however, reflect clinical practice variations. These results lack externally validity due to the non-randomised nature of this study and recruitment of the study population from one geographical area. Potential confounders such as participant comorbidities, activity level, previous injury, leg swelling and severity of KOA were not considered and can be regarded as a limitation of this work.

## Conclusion

This study is the first of its kind to report significant differences in quadriceps and patellar tendon elasticity, between individuals with KOA and asymptomatic controls. Varying elasticity was observed throughout the patellar tendon structure and may be explained by different mechanical roles. The DQT and PPT are significantly less elastic, and the DPT more elastic, in KOA individuals compared to asymptomatic older adult controls. BMI and leg circumference are associated with SE quadriceps and patellar tendon measures, with sex, BMI and OA status shown as significant explanatory correlates, within this limited population. SE detection of uncharacteristic elasticity changes may improve the understanding of the impact of in KOA and provide opportunity for early intervention and prevention of disease progression. Further longitudinal investigation including a larger sample population and correlation with clinical symptoms and biomechanical parameters including muscle strength is required to advance the clinical utility of quadriceps and patellar tendon SE in KOA.

## Supplementary Information

Below is the link to the electronic supplementary material.

Supplementary file1 (DOCX 79 KB)
