# Supplementary material for: Strain sonoelastography in asymptomatic individuals and individuals with knee osteoarthritis: an evaluation of quadriceps and patellar tendon
Source: Rheumatol Int. 2022 Aug 17;42(12):2241–51. doi: 10.1007/s00296-022-05184-3 (PMC9548467; doi:10.1007/s00296-022-05184-3)
Supplement: Supplementary file 1 — Supplementary file1 (DOCX 79 KB) [file 296_2022_5184_MOESM1_ESM.docx]

Figure 1. Association of participant age and elasticity ratio in knee osteoarthritis (KOA) group by sex.

BMI

Figure 2. Association of Body Mass Index (BMI) and elasticity ratio for the knee osteoarthritis (KOA) group.
